# Genetic Diversity, Morphometric Characterization, and Conservation Reassessment of the Critically Endangered Freshwater Snail, *Heleobia atacamensis*, in the Atacama Saltpan, Northern Chile

**DOI:** 10.3390/biology12060791

**Published:** 2023-05-30

**Authors:** Gonzalo A. Collado, Cristian Torres-Díaz, Marcela A. Vidal, Moisés A. Valladares

**Affiliations:** 1 Departamento de Ciencias Básicas, Facultad de Ciencias, Universidad del Bío-Bío, Avenida Andrés Bello 720, Chillán 3800708, Chile; 2 Grupo de Investigación en Biodiversidad y Cambio Global, Universidad del Bío-Bío, Avenida Andrés Bello 720, Chillán 3800708, Chile

**Keywords:** Atacama Desert, Chile, endangered species, freshwater gastropods, genetic clusters, isolated populations, spring snails

## Abstract

**Simple Summary:**

In this study, we implemented a multifaceted approach, coupling phylogenetic, phylogeographical and demographic analyses together with a morphological characterization to depict the genetic patterns of *Heleobia atacamensis*, an endangered species scattered in isolated and semi-isolated but dynamic habitats of the Atacama Saltpan. We focused on snails obtained from Peine and Tilomonte, two peripherical localities, which were compared with topotypes specimens. The range extension of the species also allowed us to reassess its conservation status. Molecular analyses showed that snails from Peine and Tilomonte belong to *Heleobia atacamensis*. We also discovered genetic structure in the saltpan, represented by six genetic clusters, besides morphological differences between populations. Since the observed pattern is common for other freshwater species restricted to desert aquifers worldwide, our results represent findings applicable to analogous systems. The species, listed as Critically Endangered at regional level in 2014, was reassessed as Endangered. For an eventual management plan, we suggest incorporating the genetic information obtained here.

**Abstract:**

Evaporitic ecosystems of the Atacama Desert contain a rich endemic fauna, including mollusk species. A recent study performed in the freshwater snail *Heleobia atacamensis*, endemic to the Atacama Saltpan, revealed a strong interdependence of genetic patterns with climatic fluctuations and landscape physiography. The species is currently listed as Critically Endangered at regional scale and as Data Deficient on the International Union for Conservation of Nature (IUCN) Red List. Here, we studied genetic diversity and demographic history of several populations of the species occurring on a connectivity gradient, including snails from new peripherical localities (Peine and Tilomonte), which were compared with topotype specimens. In addition, we reassessed the conservation status using the IUCN Red List categories and criteria considering species-specific idiosyncrasy. Phylogenetic and phylogeographical analyses indicated that snails from Peine and Tilomonte belong to *H. atacamensis*. We discovered significant differentiation in shell morphology, which was generally greater in geographically isolated populations. We also inferred six genetic clusters and a demographic expansion congruent with the wet periods that occurred at the end of the Pleistocene. Considering the highest risk category obtained, *H. atacamensis* was reassessed as Endangered at regional scale. Future conservation plans should consider the genetic assemblages as conservation units.

## 1. Introduction

Species extinction is a recurring event throughout the history of life on Earth, and a variety of extinction-causing factors have been documented and hypothesized, some due to natural processes, others attributed to human activities [1,2,3]. Currently, it is widely accepted, although with some variations, that there are five main causes of species extinction: habitat loss, overexploitation, invasive species, disease, and climate change [4,5,6].

The diversity of mollusks is quite high; they are the second most diverse group after the arthropods [7]. Estimates of the number of described species of mollusks vary between authors, for example, 70,000–76,000 [8], 85,000 [7] or 120,000 [9]. Of 150,300 species assessed on the International Union for Conservation of Nature (IUCN) Red List, 28% are threatened with extinction [10]. Based on MolluscaBase [11], the IUCN Red List reports 83,125 mollusk species, of which 8934 have been assessed, and 2340 declared as threatened with extinction (11%).

Freshwater snails are an important component of aquatic ecosystems worldwide, but also a group prone to extinction because such systems tend to be ephemeral, in addition to being affected by a series of threats produced by human activities that have increased in the last decades [12,13,14]. In northern Chile, saline lakes are fragile ecosystems with a particular endemism and snail diversity [15,16,17,18,19]. According to the regulations for the classification of the wild species of the Ministry of the Environment of Chile (Ministerio del Medio Ambiente de Chile), which is based on the categories implemented by the IUCN Red List, three mollusk species are listed as Critically Endangered (CR) in northern Chile: *Biomphalaria costata* (Biese), *Heleobia atacamensis* (Philippi) and *Heleobia transitoria* (Biese) [20,21,22].

*Heleobia atacamensis* is a freshwater snail endemic to Tilopozo, a small water well located at the southern end of the Atacama Saltpan in northern Chile [15]. The species is listed as Data Deficient (DD) by the IUCN Red List due to the lack of knowledge with respect to population parameters, habitat and threats [23]. However, shortly after, the species was categorized as Critically Endangered (CR) at regional level by the Chilean state based on its restricted range, the high incidences of droughts and decline in habitat quality by extraction of water for mining activities (RCE DS52/2014, Ministry of the Environment) [21]. The threat is worrying because the Atacama Saltpan contains most of Chile’s lithium reserves and all the country’s production originates from this system [24], where the mining companies occupy approximately 80 km^2^ in their operations [25]. Recent studies indicate that significant environmental degradation related to mining activity has occurred in the saltpan over the last 20 years [25,26]. In addition, spatio-temporal analyses suggest that, although the core of the saltpan is the most affected zone, the marginal zone of the system (which corresponds to the habitat *H. atacamensis* populations) would also be affected by the brine pumping [27]. Although the conservation categories of *H. atacamensis* were based on different antecedents of the species, they were mainly established considering the single population of Tilopozo. However, a greater population of this snail has been discovered in the last decades. In a phylogenetic analysis using mitochondrial *12S* and *16S* ribosomal RNA gene sequences of two snails of the genus *Heleobia* from the Atacama Saltpan, Collado et al. [28] tentatively assigned a specimen from Peine to *H. atacamensis* while another from Tilomonte was considered as the sister group of this species. In a subsequent phylogenetic analysis using the cytochrome c oxidase subunit 1 mitochondrial gene (*COI*), Collado et al. [18] also recovered *Heleobia* sp. from Tilomonte as the sister group of *H. atacamensis*, although specimens from Peine were not included. More recently, Valladares et al. [29] used a multilocus approach (mitochondrial and nuclear sequences) to analyze samples of *H. atacamensis* from Tilopozo and other seven undescribed *Heleobia* populations located inside the Atacama Saltpan, recovering the eight populations in a monophyletic group as part of a larger clade also containing other congeneric species from the Chilean Altiplano and the Atacama Desert. In addition, based on microsatellite markers, they detected a marked genetic structure and a high level of fragmentation among populations of the species. However, despite the fact that Valladares et al. [29] significantly extended the range of *H. atacamensis* within the Atacama Saltpan, the snails from the peripherical towns of Peine and Tilomonte were not included.

Currently, knowing the distribution, genetic structure, and morphological variation of a species is a fundamental aspect in conservation biology, especially in vulnerable species, in addition to the identification of evolutionary independent lineages within the focus taxa [30,31,32,33,34,35]. Considering these guidelines, which will serve as baselines for other studies regarding species at high risk of extinction, the aims of the present study are to (1) examine phylogenetic relationships of *H. atacamensis* using mitochondrial markers considering congeneric species present in the South American Altiplano and Atacama Desert, (2) investigate the genetic variation and historical demography of the species including all known populations, (3) evaluate the variability of shell morphology among populations including a comparison between snails from Peine and Tilomonte with topotype specimens of the species, and (4) reassess the conservation status of the species considering the identification of Evolutionary Significant Units (ESUs).

## 2. Materials and Methods

**Figure 1:**
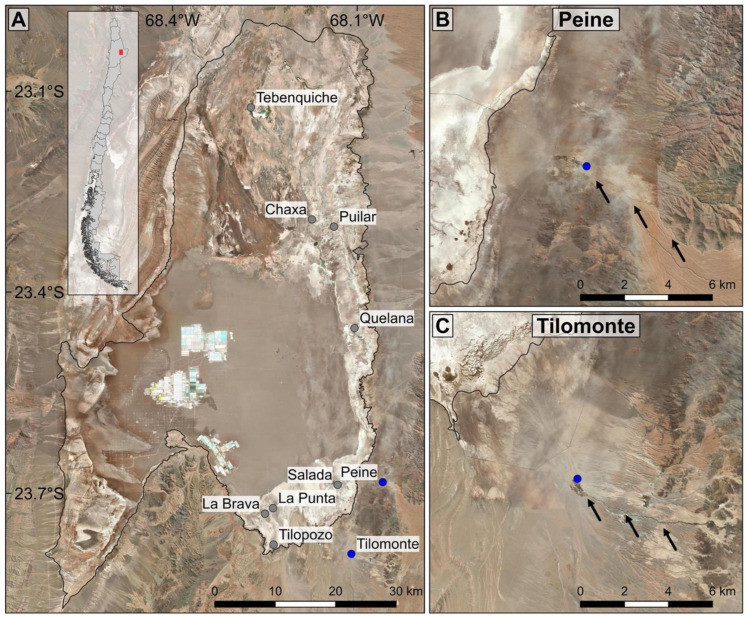
Sampling sites of *Heleobia atacamensis* in the Atacama Saltpan and adjacent localities. (**A**) Map showing the boundary of the saltpan and general view of the system. New localities are indicated in blue and previous study sites are shown in gray. (**B**,**C**) Peine and Tilomonte, respectively, showing the position and principal watercourse in each locality. The map was made using QGIS Geographic Information System v3.4.9 with ESRI world imagery (ESRI, DigitalGlobe, GeoEye, Earthstar Geographics, CNES/Airbus DS, USDA, USGS, AeroGRID, IGN, and the GIS User Community) (http://www.qgis.org, accessed on 10 January 2023). (Map: M.A. Valladares).

Live snails were collected in 2015 from Peine (23.6833° S, 68.0586° W) and Tilomonte (23.7901° S, 68.1095° W), Northern Chile (Figure 1). In each locality, 30 individuals were sampled using a sieve of 0.5 mm mesh width and stored in 70% ethanol. The shell was photographed using a Motic SMZ-168 stereomicroscope equipped with a Moticam 2000 digital camera. The soft body was isolated from the shell. The dissected opercula and radulae were put in a diluted hypochlorite solution for 3–5 min to remove organic material attached and then observed using a scanning electron microscope (SEM) Hitachi SU3500. A similar method was used for the protoconch, but in this case it was immersed for 30 min in the hypochlorite solution.

DNA extraction, PCR amplification conditions and sequencing of the *COI* gene of snails from Peine and Tilomonte were obtained following Valladares et al. [29]. Both strands of the amplified products were sequenced by Macrogen Inc. (Seoul, Korea). The sequences were edited and then aligned using the algorithm of MAFFT v7.505 [36] in the online server by Katoh and Standley [37]. In all analyses, we included sequences of different populations of *H. atacamensis* from the Atacama Saltpan [29]. This dataset considered eight sites inside the Atacama Saltpan, including Tilopozo, type locality of the species, and two outside the coast (Peine and Tilomonte) (Figure 1).

Phylogenetic relationships were examined using the maximum likelihood (ML) and Bayesian inference (BI) algorithms. In both cases, the best sequence evolution model was previously selected using PartitionFinder v2.1.1 [38]. Reconstruction by ML was performed in the program RAxML v8.0 [39], and node support was obtained by performing a bootstrap analysis of 1000 pseudoreplicates. Bayesian estimation was performed in the program MrBayes v3.2.7 [40]. The BI analysis was run three times for 50 million generations each time and the consensus tree obtained considered a burn-in of 25%. The reconstructions of ML and BI were performed in the CIPRES cluster of the San Diego Supercomputer Center [41]. In the phylogenetic reconstructions, the sequences were concatenated, and posterior analyses were implemented using a partitioned dataset. The sequences of individuals from Peine and Tilomonte were included in a comprehensive dataset depicting main lineages of *Heleobia* [18,19,28,29,42] from the South American Altiplano and Atacama Desert. Three species of *Semisalsa* Radoman were used as sister group and *Heleobops carrikeri* Davis and McKee was used as outgroup (Table S1).

For the phylogeographical and demographic analyses, the matrix generated included 123 *COI* sequences, 7 of snails from Peine, 2 from Tilomonte and 114 individuals of *Heleobia atacamensis* from eight localities inside the Atacama Saltpan [29]. This dataset included 19 sequences from individuals collected in the type locality of the species (Tilopozo). Haplotype relationships were visualized by constructing a haplotype network through the median-joining algorithm [43] using PopART v1.7 [44]. The Geneland v4.0.7 package [45] was used to determine the number of genetic groups and the spatial population structure within the species distribution. The most likely number of populations (K) was identified by performing ten independent MCMC analyses of 10^8^ iterations and thinning every 10,000 iterations. The range was limited between K = 1 and K = 10, and a burn-in of 25%.

Historical demography was studied by comparing the observed mismatch distributions [46] implemented in DnaSP v5.10.1 [47]. Harpending’s Raggedness index (rH) was calculated in Arlequin v3.0 [48] to test the unimodality of observed data. To estimate the tendency of population growth through time, we constructed a Bayesian skyline plot (BSP) in BEAST v2.5.2 [49]. This Bayesian approach incorporates uncertainty in genealogy by using MCMC integration under a coalescent model, providing information about effective population size (Ne) through time [50]. The running conditions considered 10^7^ iteration sampling parameters every 1000 steps and discarding the first 25% of steps. The analysis was implemented under a strict molecular clock and assuming an evolutionary rate of 1.7% substitutions per million years for invertebrates [51].

For morphological comparison, 20 individuals from Peine and Tilomonte were analyzed. In addition, 10 individuals from each of the eight sampling sites located inside the saltpan were included. In each case, individuals from the upper third of the size distribution of each locality were chosen. From each photograph, five shell variables were measured: shell length (SL), shell width (SW), aperture length (AL), aperture width (AW), and body whorl length (BWL). A principal component analysis (PCA) was performed to visualize the morphological variation of different populations. Since some variables violated the assumptions of normality, we analyzed the data grouping individuals by localities using the non-parametric Kruskal–Wallis (K-W) test. Subsequently, a non-parametric MANOVA test (PERMANOVA) was conducted using 10,000 permutations to compare individuals using the multivariate data and under the same grouping scheme. All statistical analyses were conducted in R v3.6.1 software [52].

Conservation status assessment of *H. atacamensis* was performed using the IUCN Red List [53] categories and criteria. For this, we used Criterion B of geographic distribution in the form of either extent of occurrence (EOO) (sub-criterion B1) and area of occupancy (AOO) (sub-criterion B2), considering (1) all localities where the species is found, (2) species “subpopulations” in the sense of IUCN Red List [53] and that (3) “The term ‘location’ defines a geographically or ecologically distinct area in which a single threatening event can rapidly affect all individuals of the taxon present” [53]. This statement is equivalent to saying that “If two or more subpopulations occur within an area that may be threatened by one such event, they must be counted as a single location” [53]. For the evaluation (3), the threats were recorded through field observations and the literature data. In this last case, we consider species-specific idiosyncrasy since *H. atacamensis* is a gill-breathing snail with direct development and therefore low vagility [54]. Geographic coordinates of all localities of the species were obtained using GPS (Global Positioning System). The evaluation was conducted loading the georeferenced localities in the software program GeoCAT (Geospatial Conservation Assessment Tool) version ß [55], which provides a preliminary conservation category based on the EOO and another on the AOO, for which a cell grid of 2 km^2^ was used. The possible categories are Critically Endangered (CR), Endangered (EN), Vulnerable (VU), Near Threatened (NT) and Least Concern (LC).

Voucher specimens were deposited in the Museo de Ciencias Naturales y Arqueología Profesor Pedro Ramírez Fuentes (MCNPPRF–CC 148–10 to MCNPPRF 148–24), Chillán, Chile and Museo de Zoología de la Universidad de Concepción (MZUC-UCCC 44212–44214), Concepción, Chile. Additional specimens are housed at Laboratorio de Malacología y Sistemática Molecular, Universidad del Bío-Bío (UBB), Chillán, Chile. *COI* sequences from Peine and Tilomonte were deposited in GenBank (OP630467-OP630474).

## 3. Results

### 3.1. Molecular Analysis

**Figure 2:**
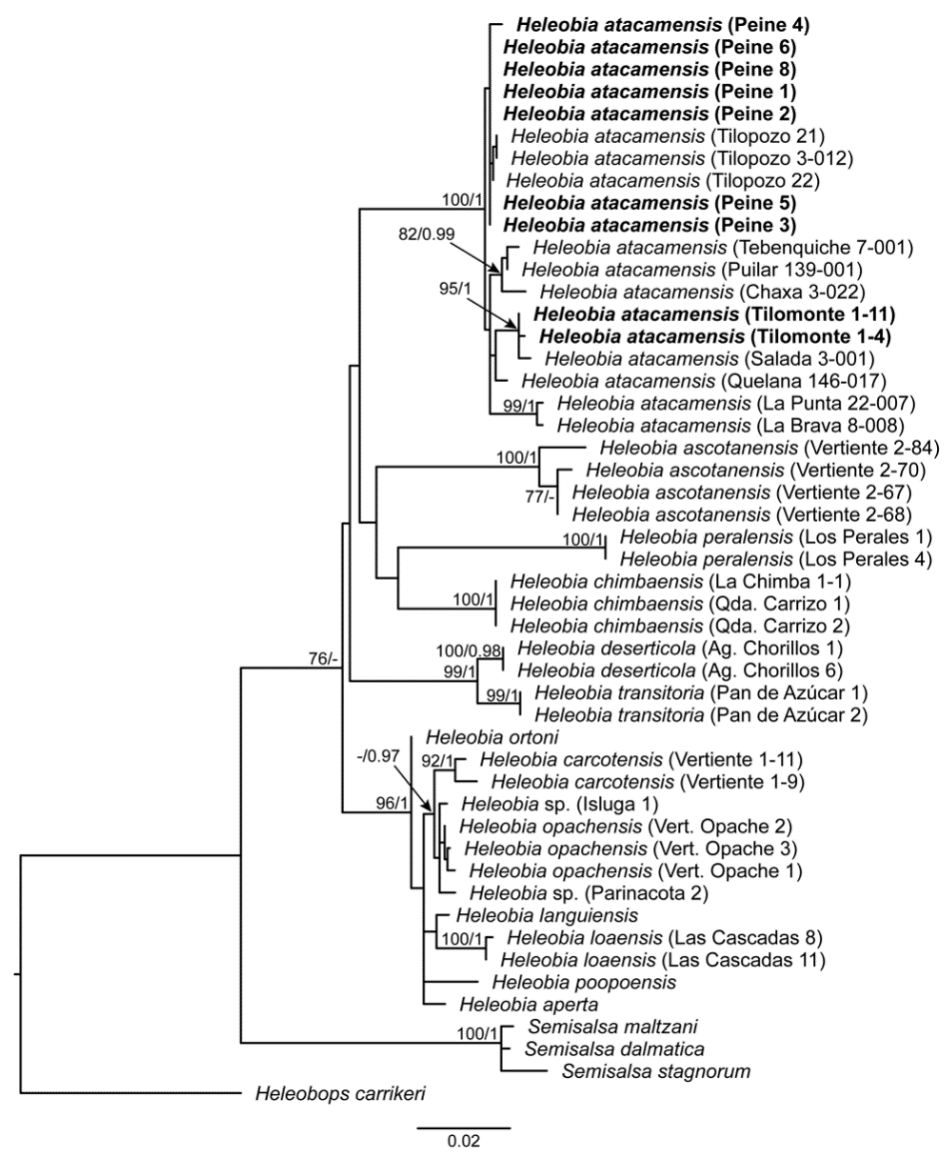
Phylogenetic relationships of *Heleobia* species of the Atacama Desert and South American Altiplano. The tree was estimated using Maximum Likelihood (ML). Node numbers indicate the support bootstrap values obtained under ML followed by posterior probabilities obtained in the Bayesian analysis. Samples from Peine and Tilomonte are highlighted in bold.

The amplification of the mitochondrial fragments (*12S*, *16S* and *COI*) used in the concatenated phylogenetic estimations produced an alignment of 1506 nucleotides in length, generating a matrix of 49 individuals. The best-fitting model of nucleotide substitution was the HKY + I + G for *12S* and *16S* genes; and GTR + I, HKY + I and GTR + G for the first, second and third codon positions of *COI*, respectively. The phylogenetic trees estimated using ML and BI were congruent, and in both analyses, samples from Peine and Tilomonte were recovered in the same clade with populations of *H. atacamensis* from inside the Atacama Saltpan (Figure 2). The *H. atacamensis* lineage presented a high node support and was differentiated from other species of *Heleobia*.

**Figure 3:**
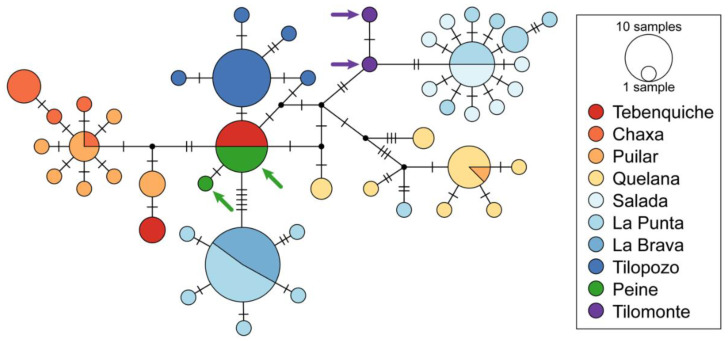
Genealogical relationships of the mtDNA haplotypes detected in *Heleobia atacamensis* (*COI* gene). Circle sizes are proportional to haplotype frequencies and colors represent the sampled localities. Arrows indicate haplotypes from peripherical localities Peine and Tilomonte.

**Figure 4:**
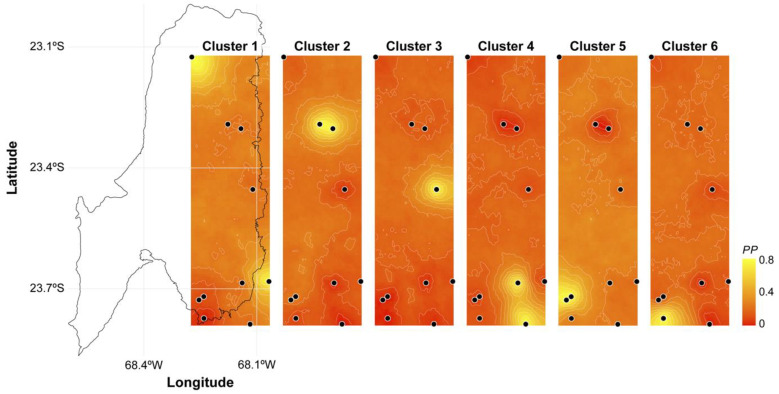
Spatial genetic structure suggested by Geneland for *Heleobia atacamensis* populations from Atacama Saltpan and new localities. Black points represent the sampling sites considered in the study. Lighter shading indicates higher probabilities of population membership.

The amplification of the *COI* gene used in the phylogeographical analyses produced a fragment of 673 nucleotides in 123 specimens examined. Sequence alignment showed a total of 55 polymorphic and 31 parsimony informative sites (nucleotide diversity, *π* = 0.011), defining 47 haplotypes (haplotype diversity, *H* = 0.928). The haplotype network (Figure 3) showed that sequences from the peripherical localities Peine and Tilomonte were recovered nested within the haplogroups of the populations from the Atacama Saltpan. The two samples from Tilomonte were recovered in two haplotypes that were not shared with any other locality. On the other hand, the individuals from Peine were associated with Tebenquiche, located in the northern section of the saltpan. The analysis of the number of genetic groups and the spatial population structure obtained with Geneland indicated the existence of six genetic clusters (posterior probability = 0.85) (Figure 4). The value of posterior probabilities associated with the definition of populations was on average 0.8. The first cluster comprised individuals from Tebenquiche and Peine. The second population contained the individuals collected in Puilar and Chaxa. The third group recovered only individuals from Quelana. The fourth cluster comprised the individuals from Salada and Tilomonte. The fifth group contained individuals from La Punta and La Brava. Finally, the sixth cluster contained only the individuals from Tilopozo.

**Figure 5:**
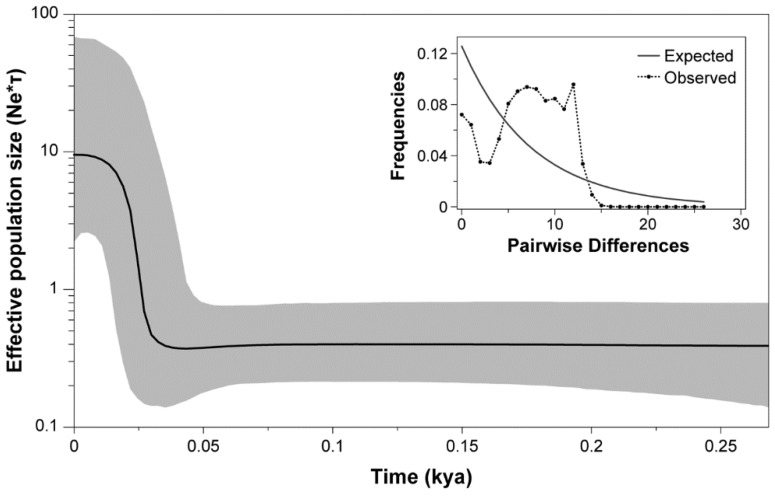
Bayesian skyline plot and mismatch distributions (inset graph) depicting the demographic history for *Heleobia atacamensis*. For the skyline plot, the black line represents mean estimates, whereas the gray area represents the 95% highest posterior density interval (HPD). For mismatch distribution, dotted line represents the observed distribution of pairwise differences and the solid black line represents the theoretical expected distribution under a population expansion model.

Demographic analyses revealed a consistent signature of recent population expansion in *H. atacamensis*. Although the mismatch distribution did not clearly show a typical unimodal and smooth distribution (Figure 5), the Harpending’s raggedness index was not significant (rH = 0.0072, *p* > 0.05). Likewise, the analysis revealed a non-significant SSD value (SSD = 0.0083, *p* > 0.05) that did not refute the demographic model of spatial expansion for the species. The historical trends in effective population size of *H. atacamensis* obtained through the Bayesian skyline plot (BSP) suggest a sudden demographic expansion that occurred in the Late Pleistocene (*c*. 25 kya) until the Early Holocene (*c*. 10 kya); after this, the effective population size remained constant (Figure 5).

### 3.2. Morphological Analyses

**Figure 6:**
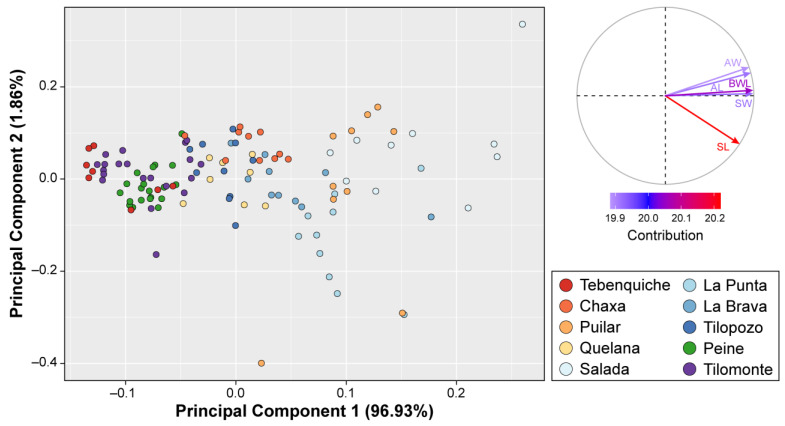
Plot of the scores on Principal Component 1 and Principal Component 2 showing morphological differences among 10 sampling sites of *Heleobia atacamensis*. To the right, the variable correlation graph is shown, and the variables are colored according to their contribution.

Together, the first two principal components explained 98.8% of the variance in shell morphology (Figure 6). While all variables had a similar contribution to the first principal component (PC1), shell length was the variable with the highest contribution to the second principal component (PC2). The PCA showed overlapping of snails of different populations across both components. However, several populations also formed clusters that differed from others mainly in the first component. In this sense, the scatter plot showed that the morphologies of the populations from Peine and Tilomonte were tightly clustered along with individuals from Tebenquiche. The results of the multivariate analyses also showed significant differences between localities (PERMANOVA pseudo-*F* = 67.113; *p* < 0.001). The five shell variables analyzed showed significant differences among localities in all comparisons: shell length (*χ*^2^ = 99.37, *p* < 0.01); shell width (*χ*^2^ = 101.99, *p* < 0.01); aperture length (*χ*^2^ = 99.68, *p* < 0.01); aperture width (*χ*^2^ = 98.81, *p* < 0001); and body whorl length (*χ*^2^ = 101.31, *p* < 0.01).

### 3.3. Conservation Status

GeoCAT calculated EOO values of 1016 km^2^ and AOO values of 40 km^2^, assigning Endangered (EN) as a preliminary threat category in both cases. Moreover, considering criterion B1 < 20,000 km² (estimated 1016 km^2^) and criterion B2 < 2000 km² (estimated 40 km^2^), more to the condition (a) regarding number of locations (equal to 10), and condition (b) of continuing decline inferred in habitat quality (iii) [51], *H. atacamensis* is categorized as Vulnerable (VU) B1ab(iii) + B2ab(iii). On the other hand, considering the six genetic clusters of the species as six subpopulations “between which there is little demographic or genetic exchange” [53], under Criterion B1 and B2 and conditions (a) and b(iii) as before, the species is reassessed as Vulnerable (VU) B1ab(iii) + B2ab(iii). However, if we consider the eight locations circumscribed only to the saltpan as one location threatened by mining activities, while Peine and Tilomonte a second location threatened by droughts (and urbanization, habitat loss), the species is classified as Endangered (EN) B1ab(iii) + B2ab(iii). As the IUCN Red List suggests that the most severe category of extinction risk must be chosen between different evaluations [53], the final Red List assessment for *H. atacamensis* is Endangered (EN) B1ab(iii) + B2ab(iii).

## 4. Systematic

Superfamily Truncatelloidea Gray, 1840;

Family Cochliopidae Tryon, 1866;

Genus *Heleobia* Stimpson, 1865.

*Heleobia atacamensis* (Philippi, 1860)

(Figure 7)

*Paludina atacamensis* Philippi, 1860: Philippi [15]: 166, Plate VII, Figure 15.

*Hydrobia atacamensis* (Philippi, 1860): Frauenfeld [56]: 575, 665.

*Littoridina atacamensis* (Philippi, 1860): Biese [57]: 172, 173, 175, Plate I, Figure a; Biese [58]: 64; Stuardo [59]: 15; Valdovinos Zarges [60]: 128; Sielfeld [61]: 3; Valdovinos Zarges [62]: 70.

*Heleobia atacamensis* (Philippi, 1860): Hershler and Thompson [63]: 50; Collado et al. [64]: 52, Figure 1; Collado et al. [28]: 6–11, Figure 2; Collado et al. [18]: 3, 6, 7; Collado et al. [19]: 278; Collado et al. [42]: 711; Collado et al. [54]: Figures 1–5.

### 4.1. Description

The morphological analysis did not provide qualitative differences between Tilopozo, Peine and Tilomonte snails; therefore, a general description is provided for the three locations.

**Figure 7:**
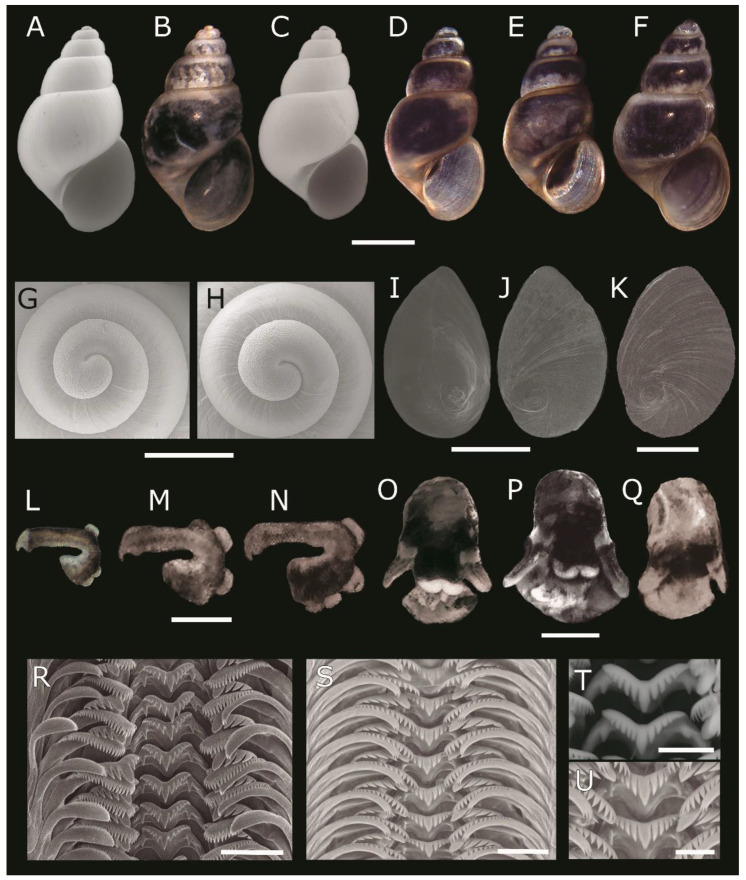
*Heleobia atacamensis* from Tilopozo, Peine and Tilomonte, Salar de Atacama. (**A**,**B**) Shell of topotype 41-UBB/MCNPPRF–CC 148–11 observed with SEM (**A**) and stereomicroscope (**B**). (**C**–**E**) Shells from Peine. (**C**) Shell of specimen 38-UBB/MCNPPRF–CC 148–23 observed with SEM. (**D**,**E**) Shells of specimens 38-UBB and 40-UBB, respectively, observed with stereomicroscope. (**F**) Shell of specimen 20-UBB from Tilomonte observed with stereomicroscope. (**G**,**H**) Protoconchs of topotype 40-UBB/MCNPPRF–CC 148–10 and 42-UBB/MCNPPRF–CC 148–12, respectively. (**I**–**K**) Opercula. (**I**) Operculum of specimen 45-UBB/MCNPPRF–CC 148–15 (inner surface) from Tilopozo. (**J**) Operculum of specimen 21-UBB (outer surface) from Tilopozo. (**K**) Operculum of specimen 2-UBB (outer surface) from Peine. (**L**–**N**). Penises. (**L**) Penis of specimen 6-UBB from Tilopozo. (**M**) Penis of specimen 31-UBB from Tilopozo. (**N**) Penis of specimen 26-UBB from Peine. (**O**–**Q**) Heads. (**O**) Head of specimen 8-UBB from Tilopozo. (**P**) Head of specimen 37-UBB from Peine. (**Q**) Head of specimen 11-UBB from Tilomonte. (**R**–**U**) Radulae. (**R**) Radula of specimen 22-UBB from Tilopozo. (**S**) Radula of specimen 30-UBB from Peine. (**T**) Central tooth of specimen 22-UBB augmented. (**U**) Central tooth of specimen 30-UBB augmented. Scale bars = (**A**–**F**): 1 mm, (**G**,**H**): 300 μm, (**I**–**N**): 500 μm, (**O**–**Q**): 1 mm (**R**,**S**): 20 μm, (**T**,**U**): 10 μm.

Shell small, conic-elongated (Figure 7A–F), thin, light brown-transparent, with closed umbilicus, smooth sculpture, deep suture. Spire high, with five convex whorls; last whorl more developed. Aperture oval, outer lip thin, frequently with a light brown line. Protoconch has less than one whorl (Figure 7G,H), 332.3 ± 11.2 μm long (n = 4), rough from the beginning to approximately the middle section and then slightly smooth, differentiated from teleoconch. Operculum paucispiral (Figure 7I–K), oval, thin, light brown in the central circular area and almost diaphanous on the outside. Penis elongated, wider at the base and anterior portion, with three to four apocrine glands on the convex side, and a small lobe near the anterior end of the concave side. The tip of the penis has a conical glans with terminal papilla (Figure 7L–N). The apocrine glands are in the proximal medial part of the organ or sometimes near the base. Color of the penis is black at the base and in the middle and convex part seen from above; the rest is gray. Foot greyish-black. Head black (Figure 7O–Q), lips white, tentacles gray at the tip and underside, black at the basal portion, separated from the head by a gray band. Radula taenioglossan (Figure 7R–U); central tooth with five–seven cusps that decrease in size from both sides of the central cusp, which is conical and much more developed.

### 4.2. Material Examined

Tilopozo (2313 m altitude), Atacama Saltpan, Northern Chile type locality.

New distributional record: Peine (2440 m altitude), Salar de Atacama basin, oasis located approximately 21 km northeast of Tilopozo; Tilomonte (2380 m altitude), Salar de Atacama basin, oasis located approximately 13 km east of Tilopozo. These two ecosystems correspond to small streams that normally do not flow into the water mirror of the saltpan, drying before reaching the coast.

### 4.3. Habitat

*Heleobia atacamensis* was collected in soft substratum or macrophytes.

### 4.4. Distribution

Tebenquiche, Chaxa, Puilar, Quelana, Salada, Peine, La Punta, La Brava, Tilopozo, Tilomonte, Atacama Saltpan basin, northern Chile.

## 5. Discussion

The phylogenetic estimations confirm the presence of *Heleobia atacamensis* in Peine and Tilomonte, previously hypothesized using rDNA sequences, although the last population was considered a candidate species of *Heleobia* [28]. These two localities correspond to records of the species outside the coast of the Atacama Saltpan. In our *COI* phylogenetic analysis, *H. atacamensis* integrated a clade together with sequences of *Heleobia ascotanensis* (Courty), *Heleobia chimbaensis* (Biese), *Heleobia deserticola* Collado, *Heleobia peralensis* Collado, Fuentealba, Cazzaniga and Valladares and *Heleobia transitoria*, all of them from northern Chile. Our results are also consistent with those of Valladares et al. [29] regarding the monophyly of *H. atacamensis* in the southern Altiplano. Several freshwater species within different taxa such as snails of the genus *Biomphalaria* Preston, fishes of the genus *Orestias* Valenciennes and frogs of the genus *Telmatobius* Wiegmann, co-distributed in the area, have also been recovered as a monophyletic group [65,66,67]. This reflects the biogeographical influence on the speciation processes of different taxa occurring at regional scale in a particular landscape.

The genetic divergence within *H. atacamensis* is consistent with studies performed in other desert spring systems, where poorly dispersing species such as snails and crustaceans show highly geographically structured populations and lineages [68,69,70,71]. We discovered that most *H. atacamensis* populations were highly genetically structured, with little evidence for migration between isolated localities as Tilopozo, Tilomonte and, to a lesser extent, Quelana. On the other hand, geographically close populations (e.g., Chaxa-Puilar, La Punta-La Brava) showed strong evidence of connectivity. The pattern detected in La Punta is also interesting since in this population, we recovered highly divergent haplotypes. This suggests that this population had some degree of gene flow with La Brava and Salada populations. This evidence was also reported in *H. atacamensis* using microsatellite markers [29], which is consistent with results obtained in other gastropods distributed in semi-isolated spring systems located in saltpans of the region [70,71]. Furthermore, both the haplotype network and the Geneland analysis showed that the populations of Tebenquiche and Peine conform a common gene pool. This finding was surprising because both localities are separated by 72.3 km. As direct migration of propagules between both localities is unlikely because the species has direct development and adults have limited vagility [64], we hypothesize the existence of passive dispersal of snails by waterfowl, a phenomenon that occurs in *Heleobia* (pers. obs.). A second explanation for this genetic similarity is a historical connection between the populations.

The inclusion of two new populations of *H. atacamensis* in the phylogeographic analyzes generated two main results. Firstly, we obtained evidence that the evolutionary history of the species would be circumscribed not only to the events that occurred within the limits of the Atacama Saltpan. Therefore, to understand the evolutionary processes and evaluate possible threats to the conservation of the species, it is necessary to carry out studies that consider all the populations of the saltpan, including Peine and Tilomonte. Secondly, the population of Tilopozo (type locality of the species) would correspond to a population differentiated from the rest, which partially differs from the results obtained by Valladares et al. [29] since the Tilopozo individuals were recovered as a genetic population with Tebenquiche, accounting for gene flow between populations.

Population demographic parameters indicate that *H. atacamensis* would have experienced a population expansion at the end of the Pleistocene, agreeing with the data indicating that wettest perennial lake interval (26.7–16.5 ky) and climatic fluctuations occurred in the Atacama Desert in that period [72,73,74]. These results reinforce the close relationship between climatic influence and demographic history in species with low vagility. In this sense, considering that the species of the genus *Heleobia* are strictly aquatic, the impact of water availability on the resilience of species that inhabit desert systems is of particular concern. This is even more important in *H. atacamensis* considering that in the saltpan, in addition to brine pumping, freshwater is extracted for mining [75], which has generated a decline in the total water storage [26].

No conspicuous qualitative differences were observed in shell morphology when comparing snails from Peine and Tilomonte with topotypes of *H. atacamensis*. However, morphometric analysis of the shell showed significant differences among populations, although many of them overlapped in the multivariate space. On the other hand, some populations including snails from Peine, Tilomonte and Tebenquiche formed cohesive clusters in bounded sectors of the morphospace. This result is noteworthy since these populations are geographically isolated and, in the case of Tilomonte, present exclusive haplotypes (although only two individuals were sequenced). Tilopozo samples also formed a morphological assemblage with Chaxa and Quelana, although less evident than in the previous examples. In this case, the genetic differentiation of the first population was also evident in all the analyzes performed, consistent with previous studies [29].

*Heleobia atacamensis* can be easily distinguished through the penis morphology of *H. ascotanensis*, *H. chimbaensis*, *H. deserticola*, and *H. transitoria*, but it is rather similar to that of *Heleobia loaensis* (Biese), *H. opachensis* (Biese) and *H. carcotensis* Collado, Valladares and Méndez, maybe reflecting evolutionary stasis since they are closely related species. However, the organ has been widely used to describe and recognize species of *Heleobia*, being of taxonomic usefulness in most cases [19,28,42,63,64,76,77,78,79,80,81,82,83]. Penis morphological differences detected previously between *H. atacamensis* and snails from Tilomonte that led to suggest the presence of a candidate species of the genus supported by a sister group relationship [28] would be the product of the low number of individuals analyzed and correspond to interspecific differences. Parasitism in *Heleobia* can also generate morphological differences in the shape of the penis [84].

In this paper, we reassessed the conservation status of *H. atacamensis* as Endangered (EN), corresponding to a higher category than Data Deficient (DD) previously listed for the species according to the IUCN Red List [23], but lower with respect to the regional classification as Critically Endangered (CR) established in Chile in 2014 [21]. However, this latter evaluation is mainly related to the number of occurrences of the species since in that year, there was only one record available. The final evaluation carried out in the present study was based on two locations and the major threats affecting the saltpan basin, mining and droughts, although other threats such as urbanization, recreation (swimming pools in Peine), agriculture, livestock, water extraction, and roads, among others, are present. As the threats and the category of high risk of extinction of *H. atacamensis* are of concern, it is necessary to implement a conservation program for the species. At present, there are environmental interpretive trails in some localities of the saltpan, with restricted access, which are under the care of the native population of the place. Apart from this, we suggest monitoring the different occurrence localities periodically, in addition to creating micro-reserves, which have been successful in other taxonomic groups [85]. Captive breeding programs can also be implemented, as in other mollusks [86,87]. *Heleobia* species, which are small, can be kept in small containers without major complications. However, a comprehensive management should consider the genetic structure of the snail, especially regarding the six genetic populations identified in the saltpan, in addition to prioritize areas for conservation based on the genetic diversity, gene flow and connectivity between systems.

For poorly investigated and endangered species, it is essential to know the number of populations and range extent they present. The new records reported in the present study are a significant finding for *H. atacamensis*, although more surveys inside and outside of the saltpan basin that could lead to the discovery of new populations of the species are required.

## 6. Conclusions

In this study, two new populations and six genetic clusters of *H. atacamensis* were identified in the Atacama Saltpan basin, constituting a baseline for an eventual management plan of this snail. We also detected morphological variation and genetic structure within the new range of the species, with signs of relatively large-scale internal migration. Further studies are needed to determine whether there are other populations of *H. atacamensis* within the basin and in other basins of the Atacama Desert and southwestern Altiplano. *Heleobia atacamensis* meets the IUCN criteria to be listed as Endangered (EN) at regional scale.

## Data Availability

All data generated by this study are available in this manuscript and the accompanying Supplementary Materials.

## Supplementary Materials

The following supporting information can be downloaded at https://www.mdpi.com/article/10.3390/biology12060791/s1; Table S1: Details and GenBank accession numbers of the additional sequences used in the phylogenetic estimations.
