# Supplementary material for: Genetic Diversity, Morphometric Characterization, and Conservation Reassessment of the Critically Endangered Freshwater Snail, Heleobia atacamensis, in the Atacama Saltpan, Northern Chile
Source: Biology (Basel). 2023 May 30;12(6):791. doi: 10.3390/biology12060791 (PMC10295427; doi:10.3390/biology12060791)
Supplement: Supplementary file 1 [file biology-12-00791-s001.zip › biology-2191493-supplementary.pdf]

Supplementary Material

Genetic diversity, morphometric characterization, and conservation reassessment of the Critically Endangered freshwater snail, *Heleobia atacamensis*, in the Atacama Saltpan, northern Chile

Gonzalo A. Collado, Cristian Torres-Díaz, Marcela A. Vidal and Moisés A. Valladares

Supplementary Table S1. Details and GenBank accession numbers of the additional sequences used in the phylogenetic estimations. Loci without available information (denoted with "--") were coded as missing data (?).

**Species**	**Identifier**	**12S**	**16S**	**COI**
Heleobops carrikeri		--	AF212902	AF213347
Semisalsa dalmatica		--	AY676119	AF367631
Semisalsa maltzani		--	--	KM213732
Semisalsa stagnorum		--	JX970535	JQ973024
Heleobia ascotanensis	Vertiente 2-84	KF285877	KF285833	KF658136
Heleobia ascotanensis	Vertiente 2-67	KF285874	KF285831	--
Heleobia ascotanensis	Vertiente 2-68	KF285875	KF285832	--
Heleobia ascotanensis	Vertiente 2-70	KF285876	--	KF658133
Heleobia carcotensis	Vertiente 1-9	KF285872	KF285829	KF658132
Heleobia carcotensis	Vertiente 1-11	KF285873	KF285830	KR816827
Heleobia chimbaensis	Qda. Carrizo 1	KF285899	KF285851	MN921125
Heleobia chimbaensis	Qda. Carrizo 2	KF285900	KF285852	MN921126
Heleobia chimbaensis	La Chimba 1-1	KF285897	KF285849	KF658141
Heleobia transitoria	Pan de Azúcar 1	KF285905	KF285856	KR870995
Heleobia transitoria	Pan de Azúcar 2	KF285906	KF285857	KR870996
Heleobia peralensis	Los Perales 1	KF285924	KF285865	MN921139
Heleobia peralensis	Los Perales 4	KF285925	KF285866	MN921141
Heleobia deserticola	Ag. Chorrillos 1	KF285907	KF285858	KR870997
Heleobia deserticola	Ag. Chorrillos 6	KF285908	KF285859	KR870998
Heleobia loaensis	Las Cascadas 8	--	KF285840	KF658164
Heleobia loaensis	Las Cascadas 11	--	KF285841	KF658167
Heleobia opachensis	Vertiente Opache 1	KF285895	--	KF658109
Heleobia opachensis	Vertiente Opache 2	--	KF285847	KF658110
Heleobia opachensis	Vertiente Opache 3	KF285896	KF285848	KF658111
Heleobia sp.	Isluga 1	KF285870	--	KF658143
Heleobia sp.	Parinacota 2	KF285869	KF285828	KF658146
Heleobia languiensis		--	--	JQ973042

Heleobia poopoensis		--	--	JQ973050
Heleobia ortoni		--	--	JQ973049
Heleobia aperta		--	--	JQ973036
Heleobia atacamensis	Tilopozo 21	KF285885	KF285835	KF658137
Heleobia atacamensis	Tilopozo 22	KF285886	KF285836	--
Heleobia atacamensis	Tebenquiche 7_001	OM494466	OM490341	OM490617
Heleobia atacamensis	Chaxa 3_022	OM494476	OM490351	OM490651
Heleobia atacamensis	Puilar 139_001	OM494497	OM490372	OM490641
Heleobia atacamensis	Quelana 146_017	OM494501	OM490376	OM490645
Heleobia atacamensis	La Punta 22_007	OM494484	OM490359	OM490494
Heleobia atacamensis	La Brava 8_008	OM494481	OM490356	OM490457
Heleobia atacamensis	Salada 3_001	OM494447	OM490322	OM490556
Heleobia atacamensis	Tilopozo 3_012	OM494487	OM490362	OM490601
Heleobia atacamensis	Tilomonte 1-4	KF285884	KF285834	KF658138
Heleobia atacamensis	Tilomonte 1-11	--	--	This study
Heleobia atacamensis	Peine 1	KF285887	--	This study
Heleobia atacamensis	Peine 2	KF285888	KF285837	This study
Heleobia atacamensis	Peine 3	--	--	This study
Heleobia atacamensis	Peine 4	--	--	This study
Heleobia atacamensis	Peine 5	--	--	This study
Heleobia atacamensis	Peine 6	--	--	This study
Heleobia atacamensis	Peine 8	--	--	This study
